# Immune Landscape of Thyroid Cancers: New Insights

**DOI:** 10.3389/fendo.2020.637826

**Published:** 2021-04-27

**Authors:** Elisa Menicali, Martina Guzzetti, Silvia Morelli, Sonia Moretti, Efisio Puxeddu

**Affiliations:** Department of Medicine and Surgery, University of Perugia, Perugia, Italy

**Keywords:** thyroid cancer, tumor microenvironment (TME), TCGA (The Cancer Genome Atlas), immunoscore, immune phenotype, immunotherapy

## Abstract

Immune system plays a key role in cancer prevention as well as in its initiation and progression. During multistep development of tumors, cells must acquire the capability to evade immune destruction. Both *in vitro* and *in vivo* studies showed that thyroid tumor cells can avoid immune response by promoting an immunosuppressive microenvironment. The recruitment of immunosuppressive cells such as TAMs (tumor-associated macrophages), TAMCs (tumor-associated mast cells), MDSC (myeloid-derived suppressor cells), TANs (tumor-associated neutrophils) and Tregs (regulatory T cells) and/or the expression of negative immune checkpoints, like PD-L1 (programmed death-ligand 1), CTLA-4 (cytotoxic T-lymphocyte associated protein 4), and/or immunosuppressive enzymes, as IDO1 (indoleamine 2,3-dioxygenase 1), are just some of the mechanisms that thyroid cancer cells exploit to escape immune destruction. Some authors systematically characterized immune cell populations and soluble mediators (chemokines, cytokines, and angiogenic factors) that constitute thyroid cancer microenvironment. Their purpose was to verify immune system involvement in cancer growth and progression, highlighting the differences in immune infiltrate among tumor histotypes. More recently, some authors have provided a more comprehensive view of the relationships between tumor and immune system involved in thyroid carcinogenesis. The Cancer Genome Atlas (TCGA) delivered a large amount of data that allowed to combine information on the inflammatory microenvironment with gene expression data, genetic and clinical-pathological characteristics, and differentiation degree of papillary thyroid carcinoma (PTC). Moreover, using a new sensitive and highly multiplex analysis, the NanoString Technology, it was possible to divide thyroid tumors in two main clusters based on expression of immune-related genes. Starting from these results, the authors performed an immune phenotype analysis that allowed to classify thyroid cancers in hot, cold, or intermediate depending on immune infiltration patterns of the tumor microenvironment. The aim of this review is to provide a comprehensive and updated view of the knowledge on immune landscape of thyroid tumors. Understanding interactions between tumor and microenvironment is crucial to effectively direct immunotherapeutic approaches in the treatment of thyroid cancer, particularly for those not responsive to conventional therapies.

## Introduction

Tumorigenesis is a multistep process that allows proliferation of mutated cells and development of neoplastic mass. Solid tumors are a heterogeneous population of different cell types including cancer cells, cancer stem cells, stromal cells (such as fibroblasts, mesenchymal stromal cells, endothelial cells, pericytes) and immune cells [such as T and B lymphocytes, natural killer cells (NKs) and tumor-associated macrophages (TAMs)]. These cells, the produced molecules (such as cytokines, chemokines, enzymes, and other soluble factors) and the extracellular matrix constitute the tumor microenvironment (TME). The interactions between tumor and TME progressively change and shape the neoplastic development also influencing the prognosis of cancer patients (1). Today it is known that for tumor survival and progression cancer cells must acquire the ability to evade the antitumor immune response (immune escape) (2). Immune escape is the last step of immunoediting, the process by which cancer cells gain the property of evading immune system (3). Thanks to immune surveillance, the first stage of immunoediting, transformed cells that accumulate gene mutations in gatekeepers or caretakers and express aberrant antigens on their surface, are recognized and eliminated by the immune system. This phenomenon can lead to the selection of those cells that have developed strategies to escape immune survaillance. The possibility of a complete cancer eradication disappears and a phase of dynamic equilibrium (immune equilibrium) is established in which immune system can only control the tumor growth and determine its continuous remodeling. Finally, the further and progressive accumulation of genetic mutations and modification in the TME allow the tumor to evade completely immune response.

Reduced immune recognition, increased resistance and development of an immunosuppressive cancer environment are the main tumor escape strategies (4). The mechanisms for implementing these strategies are different. In first instance, cancer can prevent exposure on the cell surface of tumor-specific antigens (molecules exclusively expressed on tumor cells and encoded by mutant genes) and tumor-associated antigens (shared by normal and tumor cells), in order to be less immunogenic. Secondly, cancer cells can circumvent the immune system by acting on the function of NK cells. NK cells usually kill the neoplastic clones by binding the major histocompatibility complex (MHC) class I molecules exposed by the tumor and mediating then a cytolytic response. In order to inactivate the cytolytic power of NK cells, cancer cells down-regulate the attracting-NK chemokines, such as C-X-C motif chemokine ligand (CXCL) 5, and reduce at minimal levels the exposure of MHC class I molecules on their surface (5). Accordingly, the theory of “camouflage and sabotage” was proposed (6). Camouflage is the reduction of the expression of MHC class I molecules and the escape from cytotoxic T cells activity. Sabotage is a process by which cancer cells can disrupt anti-tumor immunity through induction or recruitment of immune-suppressive myeloid cells or regulatory T cells (Tregs). Cancer cells can adopt other immunosuppressive mechanisms to create a tolerant microenvironment, such as the production of metabolic enzymes like indoleamine 2,3-dioxygenase 1 (IDO1) or arginase (ARG) or of immunosuppressive cytokines (for example interleukin (IL)-10 and transforming growth factor-β (TGF-β). Moreover, tumor cells can exploit a mechanism that T cells use to prevent the occurrence of autoimmune reactions, i.e., the expression of negative immune checkpoints such as programmed cell death ligand 1 and 2 (PD-L1/2) (7).

Focusing on thyroid tumor, this review wants to provide a comprehensive and updated view of the knowledge on the tumor microenvironment and its role in development and progression of cancer with the aim to identify strategies, based on the tumor immune context, for targeted and effective therapeutic approaches. In first instance, we will summarize the components of thyroid TME, mainly found by immunohistochemistry (IHC) and *in vitro* and *in vivo* assays. Subsequently, we will consider gene expression studies that allowed to characterize and classify thyroid tumors based on their immune signature. Finally, starting from the different types of thyroid immune phenotypes, we will provide an analysis of the potential immunotherapeutic approaches.

## Thyroid Cancer Overview

Thyroid cancer is the most prevalent endocrine malignancy and its incidence has increased over the past three decades, as indicated by epidemiological data from world cancer registers (8). This increased finding of thyroid cancers is due not only to worsening of environmental conditions (pollution, greater exposure to radiation and nutritional status) but also to the adoption of more restrictive parameters in the World Health Organization (WHO) diagnostic criteria and to the improvement of investigation techniques including cytological analysis of thyroid nodules (Fine Needle Aspiration Biopsy, FNAB) and imaging methods (9). Based on etiology, morphology, and clinical behavior, thyroid cancers are divided in well differentiated thyroid cancers (DTCs), poorly differentiated thyroid cancers (PDTCs), anaplastic thyroid cancers (ATCs), and medullary thyroid cancers (MTCs). DTCs derive from thyroid follicular cells and include papillary thyroid cancers (PTCs), follicular thyroid cancers (FTCs), and Hürthle cell carcinomas (HCCs). These tumors maintain the typical differentiation characteristics of thyroid tissue such as the ability to capture iodine, synthesize thyroglobulin, and respond to TSH (thyroid stimulating hormone). These phenotypic properties are lost by cancer cells passing from well differentiated to poorly differentiated carcinomas up to anaplastic carcinomas, which are the most undifferentiated. DTCs account for 90% of all thyroid cancers and have relatively good prognosis. About 80–85% of patients show a good response to traditional therapies that include surgery, radioiodine treatment and TSH suppressive therapy. The remaining 15–20% of patients experiences recurrence or persistence of disease, often associated with resistance to radioiodine treatment (10). PDTCs and ATCs account for about 1–5% of thyroid cancers, they are very aggressive and usually associated with a poor prognosis due to the lack of effective treatments (11). MTCs, derived from thyroid parafollicular cells, account for about 5–10% of thyroid cancer and the current treatment is limited to surgery (12).

## Genetics of Thyroid Cancer

Over time, characterization of the genomic landscape has allowed to classify thyroid tumors into molecular subtypes. In turn, this has allowed to use the tumor mutational profile for the development of targeted therapy approaches. A number of studies have characterized the presence of mutations or fusion genes in thyroid tumors using modern next generation sequencing (NGS) techniques (13–15). Mitogen-activated protein kinase (MAPK), phosphoinositide 3-kinase (PI3K), wingless/integrated (Wnt), and tumor protein P53 (p53) pathways are generally involved in thyroid tumorigenesis.

*BRAF (rapidly accelerated fibrosarcoma homolog B)* and *RAS (rat sarcoma)* mutations, or *RET/PTC* (*rearranged during transfection/papillary thyroid cancer*) rearrangements are the most common genetic alterations in DTCs. These alterations lead to the activation of the oncogenic MAPK or PI3K pathways. PTCs are usually characterized by *BRAF* mutations, including the very common *BRAFV600E* substitution, and by *RET/PTC* rearrangements. The *BRAF* driver mutation causes the constitutive activation of the MAPK pathway with loss of differentiation, tumor progression and apoptosis inhibition (16). *RET/PTC1* and *RET/PTC3* are the most common fusion forms of the chimeric oncogene *RET*, both originating from an inversion of chromosome 10. *RAS* mutations occur in PTCs with a lower prevalence and define a less aggressive subset of tumors. Conversely, the oncogenic drivers in FTCs are typically *RAS* mutations (*NRAS*, *HRAS*, and *KRAS*) and *PAX8/PPARγ (paired box 8/peroxisome proliferator activated receptor γ)* rearrangements. *RAS* mutations lead to its constitutive activation and are tumorigenesis driver mutations (15, 17, 18).

It is known that advanced thyroid tumor histotypes, such as PDTC and ATC, have an accumulation of molecular alterations compared to DTCs. The different mutational profile of the various histotypes, associated with a strong cognition of the TME, could allow for the identification of markers with diagnostic, prognostic, and therapeutic potential. Duan and coworkers (13) used a NGS panel to characterize the PDTCs and ATCs mutational pattern. Their data showed that ATCs have a higher mutation rate than PDTCs, but a lower oncogenic fusion rate. ATCs are typically characterized by *BRAF* mutation, whereas PDTCs by *RET* fusion, and *PAX8-PPAR gamma* or *ALK* (*anaplastic lymphoma kinase*) fusions. It is known that the mutational profiles of ATCs exhibit a prevalence of *TP53*, *TERT (telomerase reverse transcriptase)*, *BRAF*, and *H/K/NRAS*, whereas PDTCs more frequently show mutations in *NRAS*, *TERT*, *EIF1AX (eukaryotic translation initiation factor 1A X-linked)*, and *ATM (ataxia telangiectasia mutated)*. Similar results were obtained by Landa et al. and Chen et al. (14, 19). *TERT* promoter mutations and *TP53* mutations are considered late events of thyroid tumorigenesis and are more common in advanced tumors than in PTCs (13, 18, 20, 21). The p53 oncosuppressor normally induces cell cycle arrest, senescence, and apoptosis. When *TP53* is mutated, it triggers uncontrolled cell replication and tumor development. p53 activation is considered a final step in tumor progression. Furthermore, mutations or deletions of the *PTEN (phosphatase and tensin homolog)* tumor suppressor lead to the constitutive activation of the PI3K pathway and promote progression and invasion in PDTCs and ATCs (18, 22, 23). Moreover, a correlation between nuclear β-catenin and loss of tumor differentiation and over-expression of genes related to proliferation is known. Indeed, mutations of Wnt pathway mediators, such as *CTNNB1 (catenin beta 1)* and *Axin1*, also appear to be common in advanced thyroid carcinomas (24).

Most MTC cases present point mutations of *RET* proto-oncogene that can occur sporadically or as hereditary germline events in the multiple endocrine neoplasia syndromes (MEN2A and MEN2B) or in familial medullary thyroid cancer (FMTC) (25). A minority of sporadic MTC are caused by *RAS* mutations.

## Thyroid Cancer Microenvironment

Findings made in the field of thyroid tumor immunology support the importance of immune system in thyroid cancer. The characterization of thyroid cancer including its microenvironment is pivotal to identify the prognosis and treatment of this malignancy. Cancer immunotherapy could represent an alternative treatment for those tumors not responsive to conventional therapies such as recurrent or persistent DTC, PDTC, or ATC. To select the more appropriate immunotherapy, it is essential to consider the type and extent of thyroid cancer immune infiltration in order to prevent its failure and/or the occurrence of immunotherapy resistance.

Several researchers have tried to characterize the thyroid TME: some of them have identified the involved cell populations and soluble mediators, others have investigated the molecular mechanisms responsible for the different microenvironment profiles. Since DTCs are the most common thyroid carcinomas, many papers in the literature concern the study of TME of these tumors.

In this section, currently known components and mechanisms of thyroid TME are summarized.

### Immune Cells

**Figure 1:**
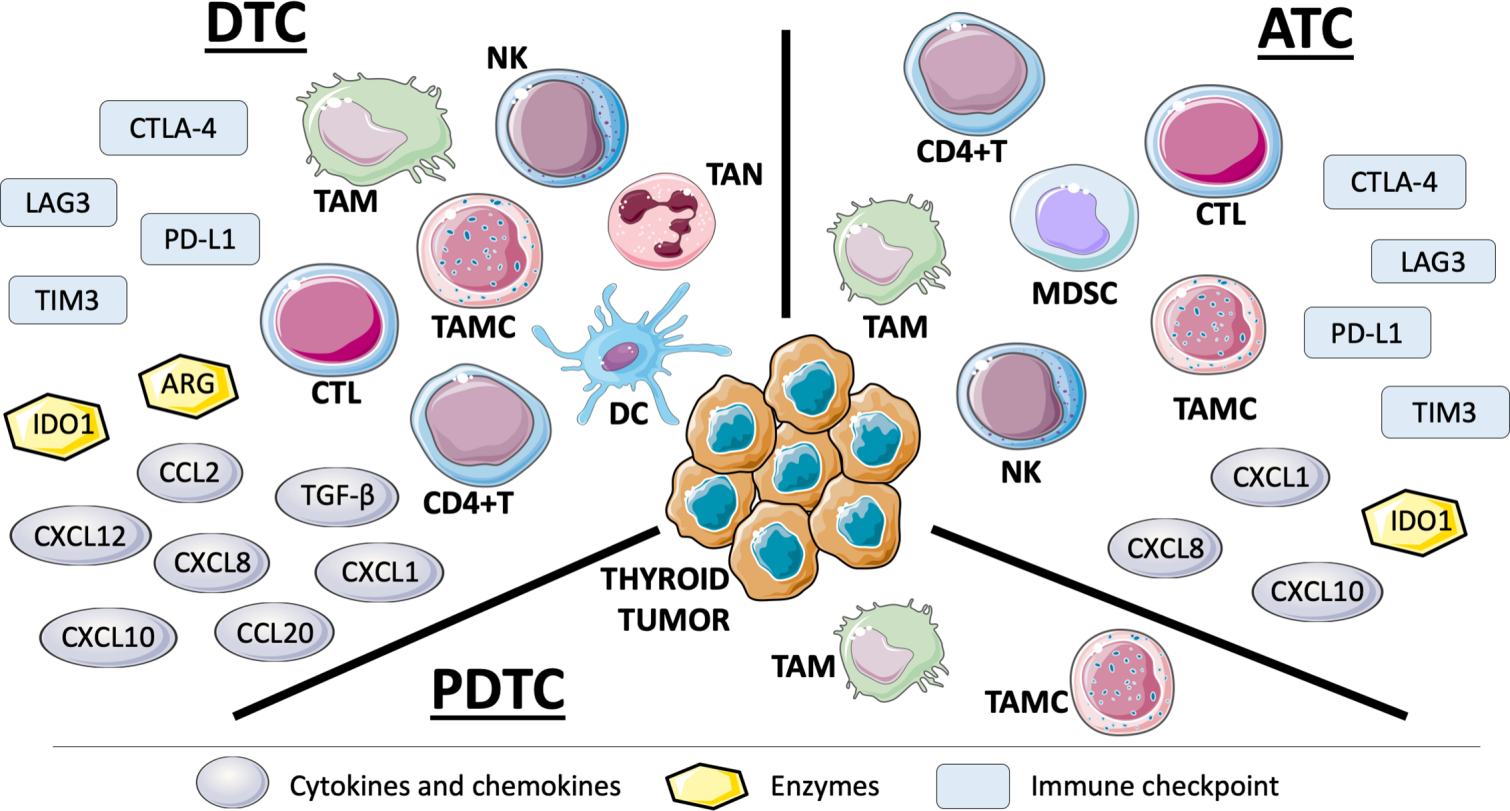
Representation of immune cells, solule mediators, and negative immune checkpoints in tumor microenvironment (TME) of thyroid tumor histotypes based on the studies carried out so far. TME of DTCs is the most frequently studied also because they are the most common thyroid carcinomas. ATCs have been shown to be richly infiltrated by cells and mediators of immune system. The few works on PDTCs display TAMs and TAMCs in their TME (see text for details).: DTC, differentiated thyroid cancer; ATC, anaplastic thyroid cancer; PDTC, poorly differentiated thyroid cancer; NK, natural killer; TAM, tumor-associated macrophage; TAN, tumor-associated neutrophil; TAMC, tumor-associated mast cell; CTL, cytotoxic T lymphocyte; DC, dendritic cell; CD4+T, CD4+ T helper cell; MDSC, myeloid-derived suppressor cell; CXCL, C-X-C motif chemokine ligand; CCL, C-C motif chemokine ligand; TGF-β, transforming growth factor-β; IDO1, indoleamine 2,3-dioxygenase 1; ARG, arginase; CTLA-4, cytotoxic T-lymphocyte associated protein 4; TIM3, T cell immunoglobulin and mucin domain 3; LAG3, lymphocyte activation gene 3; PD-L1, programmed cell death ligand 1.

The immune cell populations, mainly identified by IHC, in thyroid TME are the following (**Figure 1**).

**Tumor-Associated Macrophages (TAMs)**: they are the most represented immune cells in TME of thyroid cancer and many studies reported the correlation between TAM infiltration and clinico-pathological features of thyroid tumors. In particular, ATCs have the highest density of TAMs in TME and this correlates with poorer survival rates (26). It has been found that TAM density positively correlates with larger tumors, lymph node metastasis and decreased survival in PTC (27, 28). In PDTC, TAMs correlate with capsular invasion and extrathyroid extension (29). Moreover, Fang et al. showed that TAMs purified from human PTC can promote invasiveness of thyroid cancer cell lines through CXCL8 secretion (28). In a murine model of *BRAFV600E*-induced PTC, tumors showed a high TAM infiltration due to the increased expression of TAM chemoattractants CSF-1 (colony stimulating factor 1) and C-C motif chemokine ligand (CCL) 2 by cancer cells. In this model, TAMs displayed a M2 immunosuppressive phenotype, characterized by high production of ARG1, CCL22, and IL-10 and low levels of IL-12. Genetic and pharmacological targeting of CSF-1/CSF-1 receptor signaling impairs TAM recruitment and PTC progression (30).

**Dendritic Cells (DCs)**: tumor-infiltrating DCs usually present an immature phenotype and are unable to fully activate T cells. This is due to the low expression of co-stimulatory molecules, the high expression of inhibitory molecules and the production of immunosuppressive cytokines like IL-10 and TGF-β. DCs are not very common in thyroid cancer but it has been found that Tregs and DCs were increased in PTCs (31).

**Tumor-Associated Mast Cells (TAMCs)**: Melillo and colleagues showed that TAMC density was higher in PTCs than in normal tissue and correlated with tumor extrathyroidal extension (32). They also showed that different thyroid tumor cell lines attracted mast cells (MCs) through vascular endothelial growth factor (VEGF)-A. In turn, TAMCs released factors that induced tumor growth, survival and motility. TAMC density was higher in PTCs compared with adenomas (33). The presence of TAMCs was also analyzed in PDTCs and ATCs and it has been found that their density correlated with tumor invasiveness (34). The authors also demonstrated that MCs induced epithelial-to-mesenchymal transition (EMT) of thyroid tumor cell lines mainly through CXCL8 that activates the AKT-SLUG pathway.

**Tumor-Associated Neutrophils (TANs)**: normally involved in the early stages of inflammation, they have a controversial role in cancer. The peripheral blood neutrophil to lymphocyte ratio (NLR) was proposed as prognostic indicator for patients with tumor, according to the fact that high levels of NLR correlate with larger tumor size and higher risk of recurrence (35–38). In thyroid, although NLR was higher in tumors with poorer features, it was not significantly associated with a worse disease-free survival or higher risk of lymph node metastasis (39). Moreover, NLR failed to discriminate malignant from benign thyroid nodules (40). Galdiero and colleagues investigated tumor-associated neutrophils in thyroid cancer. They found that higher neutrophil density correlated with larger tumor size. Furthermore, thyroid cancer cell lines were able to promote neutrophil recruitment and survival through CXCL8 and GM-CSF (granulocyte macrophage-colony stimulating factor), respectively (41).

**Myeloid-Derived Suppressor Cells (MDSCs)**: these cancer-promoting cells are difficult to identify due to the lack of specific markers. Circulating MDSCs were significantly higher in ATC patients compared to healthy controls and correlated with serum levels of IL-10 (42). However, the only study that investigated thyroid tumor infiltration of MDSCs failed to demonstrate a correlation between MDSC density and clinico-pathological features of patients (43).

**Natural Killer Cells (NKs)**: essential in antiviral defense, they are also involved in immune surveillance against tumor. Two NK subgroups have been identified: cluster of differentiation (CD)56^dim^CD16^+^ NK cells have cytotoxic activity with potential ability to kill certain tumor cells; CD56^bright^CD16^−/low^ NK cells have immunosuppressive functions mediated by the release of IL-13 (44, 45). NK infiltration was significantly higher in PTC than in thyroid nodular goiter (46); circulating NKs were significantly increased in advanced thyroid cancer patients compared to healthy controls (47). Moreover, an enrichment of the less functional CD56^bright^CD16^−/low^ NK cells, expressing high levels of PD-1 (programmed cell death-1) and TIM3 (T cell immunoglobulin and mucin domain 3) exhaustion markers, has been found in advanced thyroid cancers, especially ATCs. PD-1 and TIM3 blockade reinvigorated cytotoxicity of CD56^bright^CD16^-/low^ NK cells from ATC patients, suggesting that ATC mediates NK exhaustion through PD-1 and TIM3 (47).

**CD8+ Cytotoxic T Cells (CTLs)**: CD8+ T cell infiltration correlated with favorable prognostic features in patients with DTC (43). On the contrary, the enrichment of CD8+ T lymphocytes and expression of COX2 (cyclooxygenase 2) were associated with DTC recurrence. In the majority of the tumor samples, CD8+ cells were granzyme B negative, suggesting a state of anergy (48). Moreover, a correlation between a low intratumoral CD8+/FoxP3+ (forkhead box P3) cell ratio and *BRAFV600E* PTCs has been found; markers of tumor immune suppression, namely tumor PD-L1, HLA-G (human leukocyte antigen-G), and IDO1 expression, decreased intratumoral CD8+/FoxP3+cell ratio and increased ARG1+ tumor infiltrating leukocytes were together significant predictors of tumor BRAF status (49).

**CD4+ Helper T Cells (Ths)**: they are a heterogeneous population that includes antitumoral Th1, protumoral Th2 and regulatory T lymphocytes (CD4+CD25+FoxP3+Tregs). Several papers demonstrated a correlation between tumor Treg infiltration and aggressive features of thyroid cancer. French et al. found that, in PTC, CD4+ T cell frequency correlated with tumor size while Treg frequency correlated with lymph node metastasis (50). Subsequently, the same authors proved that Tregs were enriched in tumor-involved lymph nodes and their frequency correlated with recurrent PTCs (51). Treg infiltration was also associated with more aggressive papillary thyroid microcarcinomas (PTMCs) (52). Liu and colleagues showed that higher percentage of Tregs in both peripheral blood and tumor tissue was associated with extrathyroidal extension and lymph node metastasis in PTC. Tregs were more abundant in PTCs compared to patients with hypothyroid Hashimoto’s thyroiditis but they represented only 2.5% of the total lymphocyte population (53). Interestingly, a new cell population called DN T cells (CD3+CD4-CD8-double negative T cells) was the dominant T cell population in the microenvironment of PTCs. DN T cells seemed to reduce proliferation and cytokine production of activated T lymphocytes coexisting at the tumor microenvironment (54). A poorly studied population of CD4+ T cells in thyroid is represented by CD4+IL17+T cells (Th17). They can play a protumor or antitumor role depending on the microenvironment in which they are present. Cunha and coworkers showed that Th17 infiltration was more frequent in DTCs than in non-malignant tissues (43). Another research group showed that Th17 infiltration was significantly higher in thyroid tumors than in healthy controls. Furthermore, serum levels of IL-17 of the thyroid tumor patients were positively correlated with Th17 frequency (55).

### Soluble Mediators

In thyroid cancer-related immune network, soluble mediators, mainly secreted by tumor-infiltrating immune cells but also by thyroid cancer cells, include cytokines, chemokines, angiogenic factors, and enzymes involved in amino acid metabolism (**Figure 1**).

#### Cytokines

Among cytokines found in thyroid tumors, some play an antitumor role like type I and type II interferons (IFNs) that *in vitro* induced the expression of MHC-I molecules on human thyroid cancer cell lines (56) and IL-12, a proinflammatory cytokine with strong antitumor activity (57) that has been shown to inhibit the formation of ATC in an *in vivo* model (58). Furthermore, IL-12 treatment in a mouse model of *BRAFV600E*-induced PTC significantly reduced tumor size and weight and increased mice survival (59). Other cytokines exert protumorigenic and immunosuppressive effects like IL-4, IL-10, and TGF-β. Cunha and colleagues found that IL-10 expression positively correlated with extra-thyroidal invasion and larger thyroid tumor size, suggesting a role in thyroid carcinoma progression and aggressiveness (60). Moreover, IL-4 and IL-10 have been found to increase the resistance of thyroid cancer cells to chemotherapy through the up-regulation of anti-apoptotic proteins like Bcl-2 (B cell lymphoma-2) and Bcl-xL (B cell lymphoma-extra large) (61). TGF-β up-regulation in PTCs correlated with tumor invasiveness (62). In addition, in a murine model of tumorigenesis induced by *BRAFV600E*, thyroid cells, and TAMs produced TGF-β, which was responsible for the acquisition of EMT and invasiveness of thyroid cancer cells (63).

#### Chemokines

Chemokines are a family of small molecules with chemoattractant and cytokine-like functions. They are divided in four subfamilies according to their amino acid composition, particularly to the precise configuration of the two cysteine closest to the N terminus: CXC, CC, C, and CX3C. Thyroid cells can release CXC chemokines, which include CXCL1, CXCL8, CXCL9, CXCL10, CXCL11, in basal condition and/or under the influence of specific stimuli (64). PTC and ATC cells produced high levels of CXCL1, CXCL8, and CXCL10 (65, 66). Exogenous expression of *RET/PTC1* oncogene in primary normal human thyreocytes induced CCL2, CCL20, CXCL12, and CXCL8 genes (67). Moreover, CCL20, CCL2, and CXCL8 were found up-regulated also in clinical samples of PTC, particularly those characterized by RET/PTC activation, local extrathyroid spread, and lymph node metastases, when compared with normal thyroid tissue or FTC. A study conducted in a large series of PTCs with and without associated thyroiditis showed that CCL20 and CXCL8 were significantly higher in PTC samples compared to normal thyroid, regardless of the tumor genetic background and the presence or absence of thyroiditis (68). PTC patients with high levels of CCL2 are more likely to present lymph nodes metastasis and recurrence (69). In PTC, CXCL12 expression was higher than in normal tissue and was positively related with lymph node metastasis (70). CXCL12 receptor, CXCR7, was increased in PTC and correlated with tumor progression (71).

The importance of CXCL8 in the growth and progression of thyroid cancer has been demonstrated by several studies. Bauerle and coworkers showed that CXCL8 was involved in thyroid tumor aggressiveness in an *in vivo* model of orthotopic thyroid cancer xenograft in nude mice (72). Moreover, intratumoral levels of CXCL8 were significantly higher in patients with high-risk thyroid cancer compared with those with low-risk disease. Noteworthy, Visciano and colleagues proved that CXCL8 derived from MCs *in vitro* induced EMT and stemness features in human thyroid cancer cell lines which expressed high levels of CXCL8 receptors, CXCR1 and CXCR2 (34). Fang et al. found that TAMs purified from PTCs released CXCL8 which mediated PTC cell line invasion *in vitro* and enhanced PTC metastasis *in vivo* (28). *RET/PTC1*-harboring TPC-1 and *BRAFV600E*-harboring BCPAP cell lines displayed different ability to secrete CXCL8 in basal condition and in response to TNF-α stimulation: BCPAP cells showed higher basal secretion of CXCL8 and lower response to TNF-α compared to TPC-1 (73). This suggests that differences in the genetic background determines the mechanism that regulates the release of CXCL8.

#### Angiogenic Factors

Angiogenesis, the formation of new blood vessels, is critical for tumor growth and progression (74). To date, several stimulatory and inhibitory regulators of angiogenesis have been identified (75). VEGFs, metalloproteinases (MMPs), CXCL8, and nitric oxide are some of the stimulatory regulators. Among the angiogenesis inhibitors, there are angiostatin, IFNs, and thrombospondin-1 (TSP-1). The net effect of these opposite factors determines the angiogenic behavior of the tumor. Nowadays, it is known that certain immune cells are involved in angiogenesis. For example, MCs promote angiogenesis through the release of CXCL8 (34); MDSCs secrete VEGF-A and MMP-9 (76); NK and DC cells produce VEGF-A and CXCL8 (77, 78). Factors involved in thyroid cancer angiogenesis include VEGFs and angiopoietins (Angs). However, it has not been possible to provide a more complete view of these regulators in thyroid cancer development and progression due to the lack of studies in this regard.

#### Enzymes

A pivotal role in creating a tumor-tolerant microenvironment is played by the expression of the immunoregulatory enzyme IDO1 either by the tumor cells or by the DCs that move to tumor-draining lymph nodes (79). IDO1 is a single-chain oxidoreductase that catalyzes the degradation of L-tryptophan to kynurenine, the first step in the biosynthesis of nicotinamide adenine dinucleotide. Biologically, IDO1 is involved in antimicrobial defense, in the prevention of autoimmune diseases and in maternal-fetal tolerance during pregnancy. Elevated levels of IDO1 have been observed in many human cancers, such as in ovarian cancer, in colon cancer, in breast cancer and even in thyroid cancer (80). It has been found that T cells are so sensitive to IDO1 activation that, when they are starved for L-tryptophan, they cannot divide therefore losing the ability to be activated by antigens presented to them (81). In addition, kynurenine generated by the IDO1 pathway, binding and activating the aryl hydrocarbon receptor (AhR), promotes Treg differentiation and immune suppression and fosters tumor cell survival and motility (82, 83).

A significant correlation between IDO1 expression and increased FoxP3+ tumor-infiltrating lymphocytes was found in a cohort of PTMCs (52). IDO1 levels were associated with aggressive clinico-pathologic features of the tumor such as extrathyroidal extension and multifocality, suggesting that disruption of antitumor immunity by IDO1 expression, and thus, infiltration of FoxP3+ Treg cells may contribute to tumor progression in PTMC.

Moretti and colleagues analyzed IDO1 expression in a collection of thyroid cancers (105 PTCs, 11 MTCs, and 6 ATCs). The authors showed an up-regulation of IDO1 in all the histotypes of thyroid cancer analyzed respect to the normal tissue, with mRNA expression levels highest in ATC, followed by MTC and finally PTC (84). Interestingly, the IDO1 expression increased passing from the more differentiated histotypes (PTCs and MTCs) to those characterized by greater aggressiveness (ATCs). A correlation between IDO1 expression and FoxP3+ Treg lymphocytes density in the tumor microenvironment of PTCs was observed. Moreover, the researchers, through *in vitro* co-culture experiments, provided evidences that IDO1-expressing thyroid tumor cells strongly inhibited proliferation of activated T lymphocytes and specifically increased the differentiation of Treg cells. Subsequently, Moretti and coworkers proved that RET/PTC3 induces IDO1 expression through full activation of STAT1-IRF1 pathway, demonstrating a direct link between this immunosuppressive enzyme and the oncogenic activation of *RET* in thyroid carcinoma (85).

Even insufficient concentrations of L-arginine have been shown to restrict T cell activation and proliferation. Arginase is a manganese-containing enzymes that hydrolyzes L-arginine to L-ornithine and urea in the liver urea cycle. It was initially thought to be expressed only in the liver but further studies revealed that arginase is ubiquitously expressed in many types of cells and that there are two different isoforms of this enzyme (ARG1 and ARG2) that catalyze the same biochemical reaction, but are expressed by different cells and are located in different cellular compartments (86). Increased arginase activity was found in many tumors like skin cancer, gastric cancer, cervical cancer, and thyroid cancer (87). Indeed, Cerutti and coworkers showed a moderate to intense arginase staining in the cytoplasm of tumor cells of FTC compared to healthy controls and follicular adenomas (88). Arginases may act intracellularly (cytoplasmic ARG1 and mitochondrial ARG2) and extracellularly (secreted ARG1) leading to the local depletion of L-arginine in tumor microenvironment. Moreover, ARG1 may have effects at sites distant from the TME. In detail, it has been shown to be packed into extracellular vesicles, transported over long distance and internalized by myeloid cells, for instance, in tumor-draining lymph nodes (87).

### Immune Checkpoint

Finally, immune escape may be driven by the modulation of immune checkpoints. Immune checkpoints are regulatory mechanisms able to activate or inhibit the immune response. The balance between positive and negative stimuli influences the activation of T cells and the maintenance of peripheral tolerance. In particular, negative immune checkpoints can reduce collateral tissue damage in chronic infections and prevent autoimmunity (89). Among inhibitory checkpoints, CTLA-4 (cytotoxic T-lymphocyte associated protein 4) with CD80 and CD86, PD-1 with PD-L1 and PD-L2, TIGIT (T cell immunoreceptor with Ig and ITIM domains) with PVR (poliovirus receptor), TIM3 with galectin 9, BTLA (B and T lymphocyte associated) with HVEM (herpesvirus entry mediator), LAG3 (lymphocyte activation gene 3), and VISTA (V-type immunoglobulin domain-containing suppressor of T cell activation) can be mentioned. Among stimulatory checkpoints, GITR (glucocorticoid-induced TNFR-related protein) with GITR ligand, TNFRSF9 (TNF receptor superfamily member 9) with TNFRSF9 ligand, CD40 with CD40 ligand, ICOS (inducible T cell costimulator) with ICOS ligand, and TNFRSF4 (TNF receptor superfamily member 4) with TNFRSF4 ligand should be cited (90). CTLA-4 and PD-1 are the most frequently expressed negative receptors. CTLA-4, expressed on the surface of activated lymphocytes, competes with CD28 for the binding of CD80 and CD86, expressed on antigen presenting cells (APCs). By sequestering these ligands from the CD28 co-stimulatory signal, CTLA-4 acts as a negative regulator of T cell activation (91). PD-1 is a membrane glycoprotein receptor expressed on the surface of activated T and B lymphocytes and by NK cells. By binding PD-L1 or PD-L2, PD-1 inhibits the effector function of CTLs, thus dampening the immune response (92). Tumors over-express PD-L1 or PD-L2 and exploit this mechanism as an immune escape strategy. Several studies have investigated PD-L1 expression in thyroid cancer (93). Almost all confirmed overexpression of PD-L1 in both DTC and ATC. Moreover, a significant association between PD-L1 protein and disease-free survival was observed in two large series (94, 95). In several studies, a positive correlation between PD-L1 expression and *BRAFV600E* was found (23, 49, 96). A recent meta-analysis highlighted that positive PD-L1 expression was significantly associated with poor survival in thyroid cancer patients. A significant association also emerged between increased PD-L1 expression and disease recurrence (97). Although the presence of PD-L1 in thyroid tumor microenvironment has been highly demonstrated, only few works investigated PD-L2 in thyroid cancers. Tuccilli and coworkers proved that increased PD-L2 expression correlated with *BRAFV600E* mutation and lymph node metastasis in PTC; furthermore, there was a positive correlation between PD-L1 and PD-L2 expression. However, another study failed to show an association between PD-L2 mRNA level and tumor size or lymph node metastasis in DTC (98). Recently, Giannini and colleagues demonstrated an up-regulation of inhibitory immune checkpoint mediators CD86 and CTLA-4, PD-L1/PD-L2 and PD-1, PVR and TIGIT, LAG3 and TIM3 and stimulatory immune checkpoint mediators GITR and GITR ligand, TNFRSF9, TNFRSF4, and TNFRSF4 ligand, and CD40, mainly in ATCs and at a lower extent in a subgroup of PTCs. Conversely, PDTCs did not appear to express at all these immune checkpoint mediators (99) (**Figure 1**).

Findings of a direct correlation between *RET/PTC3* and IDO1 and between *BRAFV600E* and PD-L1 suggest that different driver mutations associated with different subtype of thyroid cancer contribute to different microenvironments and differential recruitment of cells to thyroid tumors.

## From Histotype to Immune Phenotype: A New Thyroid Cancer Classification?

**Figure 2:**
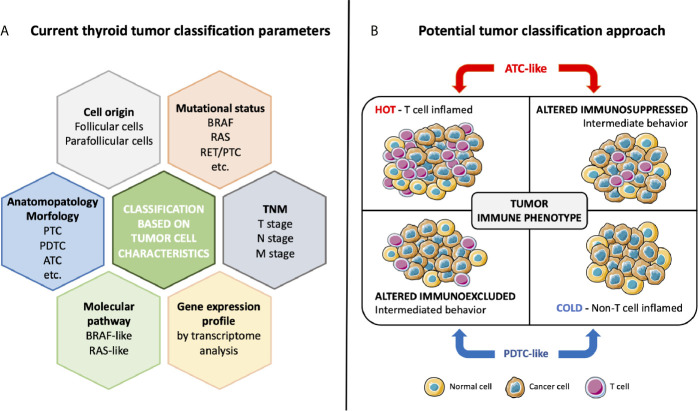
Current thyroid tumor classification based on cancer cell characteristics **(A)** and potential classification approach based on immune phenotype **(B)**. Classical classification takes into account cell origin, morphology, mutational status, altered molecular pathways, TNM stage, and signature of the tumor. The specific interaction between tumor and immune system determines the tumor immune phenotype. ATC-like tumors, presenting T cell infiltration both in the tumor center and in invasive margin or only in the tumor center, have a hot or altered immunosuppressed phenotype. Conversely, PDTC-like tumors have a cold or an altered immunoexcluded phenotype, with not or poor T cell infiltration only at invasive margin.

To improve the management of thyroid cancer patients, next to histological classification, other parameters may be taken into account including cellular origin, mutational status, molecular signaling pathway activation, differentiation, and more recently immune phenotype (**Figure 2A**).

In this section, we consider genomic and transcriptomic analyses that may contribute to define a new classification of thyroid tumors according to immune signature.

The Cancer Genome Atlas (TCGA), a study that described the genomic landscape of 496 PTCs, was the first work to suggest the usefulness of alternative classifications alongside that based on histological characteristics. This study not only extended the set of known PTC driver alterations but also identified two major subgroups of PTCs: BRAFV600E-like (BVL) PTCs and RAS-like (RL) PTCs (23). As mentioned above, PTC is a MAPK-driven cancer that has three major mutually exclusive drivers with distinct signaling consequences: *BRAFV600E*, *RAS* mutation, and *RET* fusions. Tumors driven by *BRAFV600E* do not respond to the negative feedback from ERK to RAF (since it signals as a monomer), resulting in high MAPK-signaling (100). Conversely, tumors driven by *RAS* signal *via* RAF dimers that respond to ERK feedback, resulting in lower MAPK-signaling. *RET* fusions signal through RAS, but may have an intermediate behavior between BVL and RL tumors due to the activation of other upstream signals. This differential signaling results in profound phenotypic differences. For example, expression of genes responsible for iodine uptake and metabolism are reduced in *BRAFV600E* tumors, in contrast to the *RAS* mutated tumors in which expression of these genes is largely preserved (101). Consequently, BVL-PTCs result in predominantly less differentiated tumors; conversely, RL-PTCs result in highly differentiated tumors. The authors’ conclusion is that RL-PTCs and BVL-PTCs are fundamentally different in their genomic, epigenomic, and proteomic profiles; a BRAFV600E-RAS score (BRS), ranging from −1 to +1 with BVL-PTCs being negative and RL-PTCs being positive, has been developed to quantify the extent to which the gene expression profile of a given tumor is similar to one of two profiles (14).

An integrated study conducted using TCGA data was published in 2018 and consisted of a huge immunogenomic analysis of over 10,000 cancers of 33 different types. The authors identified six immune subtypes (from C1 to C6): wound healing, IFN-γ dominant, inflammatory, lymphocyte depleted, immunologically quiet, and TGF-β dominant. These subtypes were characterized by differences in macrophage or lymphocyte signatures, Th1:Th2 cell ratio, extent of intratumoral heterogeneity, aneuploidy, extent of neoantigen load, overall cell proliferation, expression of immunomodulatory genes, and prognosis (102). Thyroid cancer, in particular PTC, was included in the Inflammatory subtype, characterized by balanced macrophage:lymphocyte ratio, high Th1:Th2 ratio, low cell proliferation, low intratumoral heterogeneity, low levels of aneuploidy and overall somatic copy number alterations and high expression of genes indicative of Th17 differentiation.

Zhao and colleagues, combining TCGA data and Estimation of Stromal and Immune cells in Malignant Tumor tissues using Expression data (ESTIMATE) datasets identified differentially expressed genes in thyroid cancer microenvironment (103). They found 793 differentially expressed genes identified to be associated with immune score and stromal score. These score values were obtained from the ESTIMATE website that predict infiltration of immune cells and stromal cells in TME. Furthermore, the authors, using these differentially expressed genes, constructed protein-protein interaction (PPI) network and conducted functional enrichment analysis. Thirty genes were selected as hub genes. Kyoto Encyclopedia of Genes and Genomes (KEGG) pathway analysis demonstrated that these genes were mainly associated with immune response. As stated by the authors themselves, a limitation of this study is that it is based on bioinformatics analysis only.

More recently, some researchers considered the Immunoscore, in addition to the TCGA results. Previously proposed by Galon to quantify immune contexture and predict prognosis in colon cancer (104), Immunoscore has also been exploited in thyroid. The Immunoscore is based on the quantification of two lymphocyte populations (usually CD3+ and CD8+ cells) presented at the center of tumor (CT) and invasive margin (IM). These parameters provide a scoring system ranging from Immunoscore 0 (I0), having low densities of both cell types in both regions, to Immunoscore 4 (I4), characterized by high densities of both cell populations in both regions (104). In colon cancer, Immunoscore has been shown to possess a greater prognostic significance than the American Joint Committee on Cancer/Union for International Cancer Control (AJCC/UICC) TNM classification system, based on age of the patient at the time of diagnosis, extent of primary tumor (T), presence of regional lymph node metastasis (N) and distant metastasis (M).

A study, performed using mRNA transcriptome data obtained by TCGA, evaluated the Immunoscore of PTCs to characterize the immune landscape (105). Authors demonstrated that Immunoscore has a negative correlation with thyroid differentiation score (TDS). In detail, PTCs with *BRAFV600E* mutation had a high grade of Immunoscore and a low TDS. PTCs were also characterized by a high score of myeloid cells, B-cells, and Tregs. The same study pointed out that immunosuppressive markers such as CTLA-4, PD-L1, and HLA-G correlated positively with the *BRAFV600E* mutation.

Looking at non-differentiated tumors, the literature suggests that there is a positive correlation in PTCs and PDTCs between TDS and BRS (23). In particular, PDTCs with *BRAF* mutation showed a decrease of TDS compared to PDTCs with *RAS* mutation. In ATCs, the correlation is lost as these tumors are deeply undifferentiated, independently of their driver mutations (14).

Recently, a paper demonstrated a new way to stratify thyroid tumors based on immune-related gene expression (99). Giannini and coworkers (99) analyzed the immune expression profile in a collection of thyroid carcinomas (25 PTCs, 14 PDTCs, and 13 ATCs) and normal thyroid (7 NT), using the innovative NanoString Technology and its nCounter PanCancer Immune Profiling Panel (106). The Nanostring analysis revealed two different clusters of tumors. One cluster characterized by all ATCs, and a part of PTCs with a high regulation of immune-related genes. The other cluster encompassed all PDTCs and the remaining PTCs with low upregulation of immune-related genes. The NT samples clustered in the latter group. Interestingly, analysis of *BRAF*, *RAS*, and *TERT* mutations revealed that the clustering did not depend on the cancer genotype. Furthermore, tumor mutational burden (TMB) showed no significant differences between the two clusters. The expression levels of genes specific for immune cell populations indicated that the TME of ATCs was richly infiltrated by TIL (tumor infiltrating leukocytes), including high density of macrophages and exhausted CD8+ T cells. Conversely, PDTCs displayed a TME poorly infiltrated by TIL. PTCs had an intermediate behavior between ATCs and PDTCs. The authors evaluated by IHC immune cell densities and, based on the Immunoscore classification proposed by Galon and colleagues in colon cancer, they suggested a new classification of thyroid cancer (**Figure 2B**). Focusing on immune infiltration, Galon et al. suggested four tumor categories: hot, altered-excluded, altered-immunosuppressed and cold (107). Hot tumors are characterized by a high degree of T cell and CTL infiltration both in CT and in IM of tumor; they present the higher Immunoscore, with a consistent activation of immune checkpoints PD-1, CTLA-4, TIM3, and LAG3 and a consequent impairment of T cells functions. Altered-immunosuppressed tumors have poor, albeit not absent, T cell and CTL infiltration with intermediate Immunoscore; they present soluble inhibitory mediators (TGF-β, IL-10, and VEGFs), immune suppressive cells (MDSCs and regulatory T cells) and T cell checkpoints (PD-1, CTLA-4, TIM3, and LAG3). Altered-excluded tumors display no T cell infiltration inside the cancer but at the invasive borders with an intermediate Immunoscore. It is possible to observe an activation of oncogenic pathways, a fine regulation of tumor microenvironment, aberrant vasculature, and/or stroma and hypoxia. These characteristics prevent the infiltration in the core of the tumor, so the immune response remain blocked at invasive margins. Finally, tumors with the lowest Immunoscore, cold tumors, are characterized by an absence of T cells within the tumor and at the tumor edges, low tumor mutational burden, poor antigen presentation and intrinsic insensitivity to T cells. In thyroid, Giannini and coworkers (99), on the basis of Nanostring results and Immunoscore, obtained by CD3+ leukocyte quantification, identified two main phenotypes: ATC-like and PDTC-like. ATC-like included tumors characterized by rich T cell infiltrate (hot and immunosuppressed tumors), high expression of CCL2, CCL3, CCL4, CCL5, CXCL9, and CXCL10 and high expression of positive and negative immune checkpoints. PDTC-like encompassed tumors with poor T cell infiltrate (cold and immunoexcluded tumors) and low expression of chemokine and immune checkpoint molecules. Conversely, PTCs displayed a mixed immunological behavior, with about 50% of samples presenting an ATC-like immune phenotype and the remaining 50% a PDTC-like immune phenotype.

In addition, the authors noted in ATCs a high density of macrophages and hypothesized that the macrophages may be M2-TAMs based on the upregulation of cytokines considered responsible for the recruitment and polarization of macrophages. The literature confirms that TAMs, macrophages that reside in the tumor microenvironment, are mainly M2 cells (108). Consistently, Landa and coworkers (14) highlighted the infiltration of M2 macrophages in ATCs compared to PDTCs. In ATCs, 50% of nucleated cells are represented by TAMs. The high density of M2-TAMs is related to aggressiveness and invasiveness, making TAMs a prognostic biomarker (29, 109).

## Potential Immunotherapeutic Approaches Based on Immune Phenotype of Thyroid Cancer

Immunotherapy has started to become a promising therapeutic option also for advanced thyroid carcinomas that are refractory to conventional treatments. It comprises a group of innovative therapies that acts by redirecting immune response against the tumor. In the case of richly infiltrated tumors, the goal of immunotherapy is to reactivate immune cells in order to restore their ability to recognize and destroy neoplastic cells. Conversely, in the case of poorly infiltrated tumors, immunotherapy should promote the trigger of an effective immune response. Characterization of the TME and understanding the mechanisms underlying the different immunological behavior of thyroid tumors is essential to guide the choice of the most suitable immunotherapeutic approaches.

ATC-like tumors (hot and altered immunosuppressed) could benefit from therapies that combine inhibitory immune checkpoints blockade and activating immune checkpoints stimulation eventually coupled to the inhibition of TAMs. Conversely, in the case of PDTC-like tumors (cold and altered immunoexcluded) that are characterized by the absence of T cell inflammation and of a rich immune infiltration, an approach to prevent immunotherapy failure is essential. In first instance, strategies for T cell recruitment and priming in the TME of these tumors should be foreseen. Secondly, immunotherapeutic approaches considered for infiltrated cancers should be included, in order to activate an effective anti-tumor immune response.

Immune checkpoint inhibitors, adoptive cell therapy, and cancer vaccines are some of the main immunotherapeutic strategies available for the treatment of thyroid cancers.

### Immune Checkpoint Inhibitors

This therapy uses monoclonal antibodies (mAbs) directed against immune checkpoints, such as anti-CTLA-4 monoclonal antibodies (i.e., ipilimumab), anti-PD-1 monoclonal antibodies (i.e., pembrolizumab and nivolumab) and anti-PD-L1 monoclonal antibodies (i.e., atezolizumab, durvalumab, and avelumab). The use of mAbs can lead to decrease proliferation, inhibit angiogenesis, promote apoptosis, and stimulate the immune system to destroy cancer cells. To date, there are no immune checkpoint inhibitors (ICIs) approved for the treatment of advanced thyroid cancers (110) but several clinical trials are ongoing to develop an appropriate treatment for this type of cancer. Noteworthy, in a patient with ATC resulted *BRAFV600E* and PD-L1 positive by NGS and IHC, sequential treatment with BRAF inhibitor vemurafenib and nivolumab led to a substantial regression of the tumor, with a complete radiographic and clinical remission 20 months after the beginning of the treatment (111). In the context of immune checkpoint inhibition, an alternative therapeutic strategy may be represented by small molecule inhibitors. Small molecule inhibitors, unlike mAbs, can interact, not only with surface receptors, but also with molecular targets inside the cell. mAbs often trigger significant side effects like autoimmune reactions. Differently, small molecules are not immunogenic and may inhibit the survival and proliferation of cancer cells with less side effects. This makes small molecules a promising therapeutic strategy.

### Adoptive Cell Therapy

It is a kind of immunotherapy based on the recruitment of immunocompetent cells isolated from cancer (112). There are two approaches to obtain adoptive cell therapy (ACT): in the first one, immune cells are expanded *ex vivo* and then inoculated into the patient; these cells can directly attack tumor or stimulate the immune system response; in the second one, T cells are engineered to become CAR-T (chimeric antigen receptor-T cell) able to identify and kill cancer cells (113). CAR-T directed against the ICAM-1 (intercellular adhesion molecule-1), found overexpressed in advanced metastatic thyroid cancers, has been proved to have therapeutic efficacy in animal models bearing ATC patient-specific tumors. ICAM-1 CAR-T cell administration killed tumor cells resulting in long term remission and significantly improved survival (114).

### Cancer Vaccines

Another important form of immunotherapy, based on the use of tumor-specific antigens, is represented by cancer vaccines. Many approaches are known today, such as the use of DCs, T cells or other immune system cells. The study of neo-antigens of tumor, to be used in vaccine-therapy, seems to be more promising in ATCs than in well differentiated tumors due to the presence of an increased mutational burden (14). The New-York Esophageal Squamous Carcinoma 1 (NY-ESO-1), belonging to the cancer/testis antigen family, is considered an important target for tumor vaccine immunotherapy. In an orthotopic mouse model with BCPAP cells, 5-aza-2’-deoxycytidine treatment induced an increased expression of NY-ESO-1, suggesting that thyroid cancer cells can be stimulated to express immune antigens recognized by TCR-based immunotherapies (115). In addition, DC vaccination loaded with tumor lysate showed a promising effect in MTC patients. Mature DCs obtained from patients’ peripheral blood monocytes and injected into lymph nodes induced a positive immunological response with a reduction of lesions in about the 50% of cases (116).

### Inhibition of TAMs

An exciting therapeutic approach for advanced thyroid tumors seems to be the recruitment inhibition of TAMs, largely present in ATCs. Several studies are investigating the recruitment block of protumoral M2-TAMs and their repolarization into antitumoral M1-TAMs. The overexpression of CSF-1, involved in the recruitment of TAMs at tumor level, correlates positively with thyroid tumor aggressiveness (117). Moreover, CCL2 stimulates TAM migration and differentiation (118). Thus CSF-1 and CCL2 inhibition may represent a hopeful immunotherapeutic approach.

## Conclusions

Understanding the interactions between tumor and immune system is instrumental to identify new molecular targets, novel immunotherapeutic approaches, and new efficacy biomarkers that may improve immunotherapy for thyroid cancer.

The information so far available supports a model of tumor progression in which genetic mutations play a role in driving initiation and progression of thyroid carcinomas by deregulating growth, resistance to apoptosis, cell survival, angiogenesis and stimulating invasion, metastasis, and dedifferentiation. The microenvironment also influences tumor development and clinical outcome. The interaction between cancer cells and tumor microenvironment depends on both specific genetic events, which induce cytokine and chemokine expression, and other factors, such as the state of patient’s immune cells influenced by environmental features including gut microbiome. In the perspective of a continuous evolution in the relationship between tumor and immune system, the combination of dedifferentiation signals with a rich, antigen-mediated, interaction between tumor cells and the microenvironment, also maintained by a high TMB, directs progression mainly toward a “hot pathway” leading to the development of T cell infiltrated primary ATCs and ATC-like tumors (hot and altered–immunosuppressed tumors). Conversely, the combination of dedifferentiation signals with a poor interaction between tumor cells and the microenvironment essentially drives a “cold pathway” leading to the development of not or poorly T cell infiltrated PDTCs and PDTC-like tumors (cold and altered–excluded tumors). Immunotherapeutic approaches will have to be different according to the tumor immune phenotype. In the case of tumors following “hot pathway,” inhibitory immune checkpoint blockers possibly combined with soluble factor inhibitors and immunosuppressive cell depletion could be adopted, in order to restore an effective antitumor response. Instead, in tumors following “cold pathway,” immunotherapy should be accompanied by other strategies able to induce immunogenic cell death, such as radiotherapy, chemotherapy and targeted therapy, in order to attract immune cells to the tumor microenvironment and then activate them to kill cancer cells.

Further studies will be needed to completely characterize thyroid TME and to clarify the mechanisms that determine why a tumor possesses a T cell inflamed or non T cell inflamed microenvironment. The availability of this knowledge will contribute to the development of personalized therapeutic solutions to counteract failure and/or resistance to immunotherapy.

## Author Contributions

EM, MG: study concept, searching and literature review, writing the manuscript. SiM: critical review. SoM, EP: study supervision, revision of the final version of the manuscript. All authors contributed to the article and approved the submitted version.

## Funding

This study was supported by a grant from the Ministero dell'Istruzione, dell'Università e della Ricerca (MIUR - Prin 2017 cod. 2017YTWKWH-003).

## Conflict of Interest

The authors declare that the research was conducted in the absence of any commercial or financial relationships that could be construed as a potential conflict of interest.
